# Effects of iron on the growth, biofilm formation and virulence of *Klebsiella pneumoniae* causing liver abscess

**DOI:** 10.1186/s12866-020-01727-5

**Published:** 2020-02-18

**Authors:** Tao Chen, Guofeng Dong, Siqin Zhang, Xiucai Zhang, Yajie Zhao, Jianming Cao, Tieli Zhou, Qing Wu

**Affiliations:** 1 Department of Clinical Laboratory, The First Affiliated Hospital of Wenzhou Medical University, Wenzhou, Zhejiang Province China; 2 Xiangyang NO.1. People Hospital, Affiliated Hospital of Hubei University of Medicine, Xiangyang, Hubei Province China; 3 School of Laboratory Medicine and Life Sciences, Wenzhou Medical University, Wenzhou, Zhejiang Province China

**Keywords:** Iron, *Klebsiella pneumoniae*, Growth, Biofilm, Virulence

## Abstract

**Background:**

*Klebsiella pneumoniae* is considered the most clinically relevant species of Enterobacteriaceae, known to cause severe infections including liver abscesses. To the best of our knowledge, a large proportion of iron in the human body is accumulated and stored in the liver. We hypothesize that increased iron availability is an important factor driving liver abscess formation and we therefore aim to understand the effects of iron on *K. pneumoniae* causing liver abscesses.

**Results:**

All tested *K. pneumoniae* clinical isolates, including those isolated from liver abscesses and other abdominal invasive infection sites, grew optimally when cultured in LB broth supplemented with 50 μM iron and exhibited the strongest biofilm formation ability under those conditions. Decreased growth and biofilm formation ability were observed in all tested strains when cultured with an iron chelator (*P* < 0.05). The infection model of *G. mellonella* larvae indicated the virulence of liver abscess-causing *K. pneumoniae* (2/3) cultured in LB broth with additional iron was significantly higher than those under iron-restricted conditions (*P* < 0.05). The relative expression levels of the four siderophore genes (*iucB*, *iroB*, *irp1*, *entB*) in *K. pneumoniae* strains isolated from liver abscesses cultured with additional iron were lower than those under iron-restricted conditions (*P* < 0.05).

**Conclusions:**

It is suggested by our research that iron in the environment can promote growth, biofilm formation and enhance virulence of *K. pneumoniae* causing liver abscesses*.* A lower expression of siderophore genes correlates with increased virulence of liver abscess-causing *K. pneumoniae*. Further deeper evaluation of these phenomena is warranted.

## Background

*Klebsiella pneumoniae* is considered the most clinically relevant species of *Enterobacteriaceae*, known to cause both community-acquired and nosocomial infections, including liver abscesses, pneumonia, urinary tract infections and bacteremia worldwide [1]. In the past two decades, a distinct hypervirulent variant of *K. pneumoniae*, characterized by its hypermucoviscous phenotype, was firstly isolated from liver abscesses in Asia and has emerged as a clinically significant pathogen responsible for highly invasive infections [2]. Unlike classical *K. pneumoniae,* hypervirulent *K. pneumoniae* (hvKp) can spread from the original site of infection to other organs. Once invasive dissemination occurs, patients often suffer severe and irreversible refractory sequelae, such as blindness and central nervous system damage [2, 3]. The conditions of patients infected with hypervirulent *K. pneumoniae* causing liver abscess are serious, posing a great threat to public health and has attracted the attention of clinicians.

The pathogenicity of *K. pneumoniae* mainly arise from various virulence factors which allow it to overcome innate host immunity and to maintain infection in a mammalian host. The main virulence factors that play an important role in pathogenicity are capsular polysaccharide, lipopolysaccharide, pili and siderophores [4]. Therein, K1 and K2 capsular types are considered to be highly pathogenic to human [5]. *rmpA* is an important activator of capsular production, resulting in the formation of hypermucoviscous phenotype and enhancement of virulence, while aerobactin (*iucB*), yersiniabactin (*irp1*), salmochelin (*iroB*) and enterobacterin (*entB*) are the four siderophores of *K. pneumoniae* [6, 7]. Therefore, understanding the virulence characteristics of *K. pneumoniae* and taking appropriate measures are critical for clinical assessment, controlling the prognosis and reducing mortality risk of patients with *K. pneumoniae* infection.

It is recognized that most organisms require iron for a variety of metabolic and informational cellular pathways [8]. However, ferric ion (Fe^3+^) and its derivatives are poorly soluble, and cannot be utilized directly by most organisms. It is essential to synthesize and secrete siderophores to meet the demand for iron required for bacterial growth and metabolism. To repress microbial growth, iron availability within the human body is strictly limited [9]. One notable exception is the liver, in which iron concentration is used for assessing body iron stores [10]. These increased iron levels probably promote growth of *K. pneumoniae* in the liver and provide an advantage for liver abscess formation. Therefore, our study was designed to evaluate the effects of iron on the growth, biofilm formation and virulence of liver abscess-causing *K. pneumoniae*.

## Results

### Antimicrobial susceptibility testing

Five *K. pneumoniae* isolates (FK3065, FK3170, FK3226, FK3992, FK4003) were sensitive to conventional antimicrobials (levofloxacin, ciprofloxacin, cefepime, ceftazidime, imipenem, gentamicin, amikacin, tobramycin, ampicillin/sulbactam, piperacillin/tazobactam, trimethoprim/sulfamethoxazole, aztreonam, cefotetan, ertapenem, ceftriaxone). However, FK3087 was resistant to ampicillin/sulbactam, levofloxacin, ciprofloxacin, gentamicin and trimethoprim/sulfamethoxazole.

### Effect of iron on growth and biofilm formation of the *K. pneumoniae* isolates

**Fig. 1:**
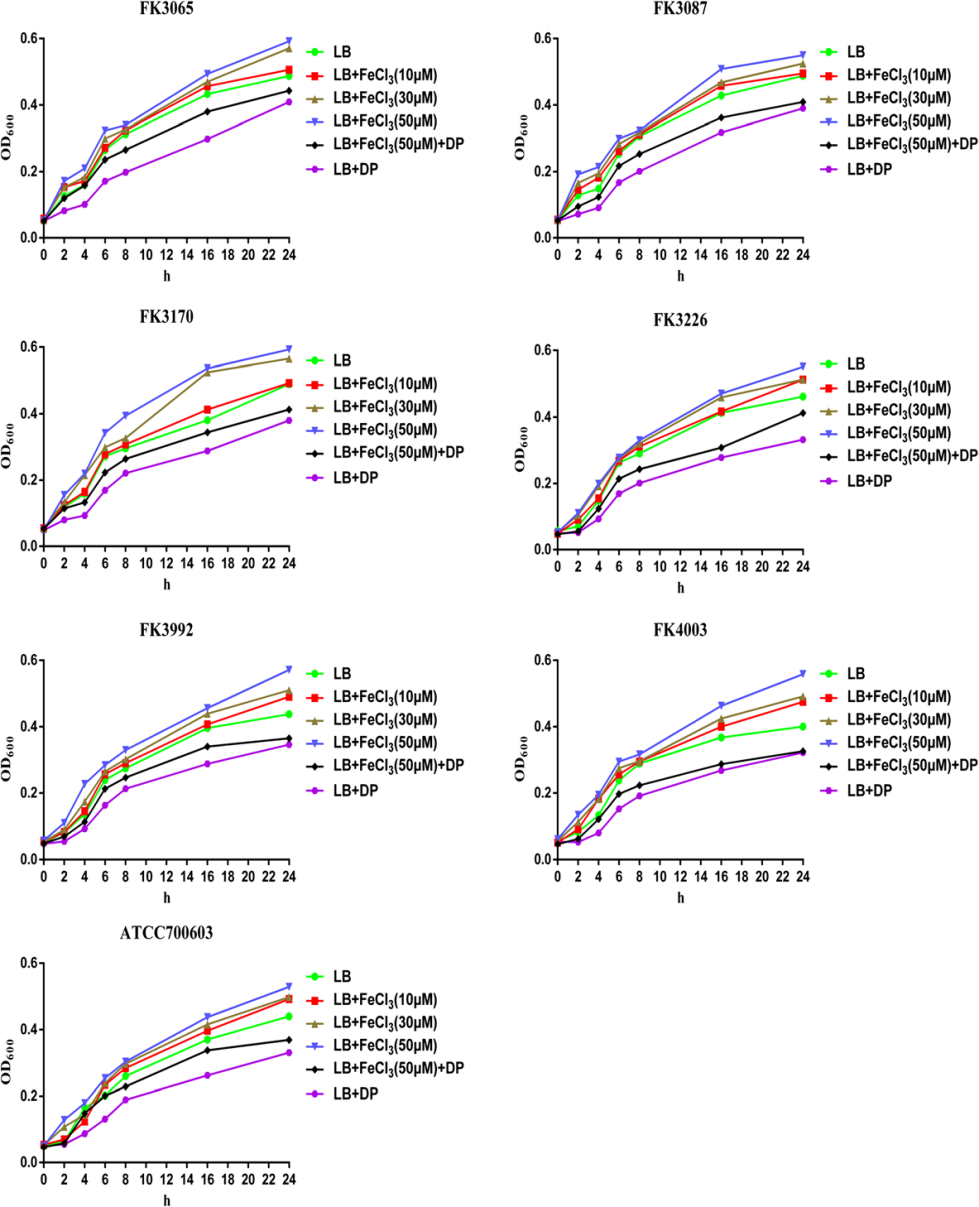
The growth curves of *K. pneumoniae*. All *K. pneumoniae* isolates grew optimally in LB broth containing 50 μM iron, superior to the growth in LB broth containing 30 μM, 10 μM, and 0 μM iron or 200 μM iron chelator +(−) 50 μM iron(*P* < 0.05). DP: 2,2′-Dipyridyl, added as an iron chelator. LB + 200 μM iron chelating agent group was used as control

All *K. pneumoniae* isolates grew optimally in LB broth containing 50 μM iron, superior to the growth in LB broth containing 30 μM, 10 μM, and 0 μM iron, separately (*P* < 0.05). The growth of all tested strains in the iron-restricted environment caused by the iron chelating agent was worse (*P* < 0.05) (Fig. 1).

**Fig. 2:**
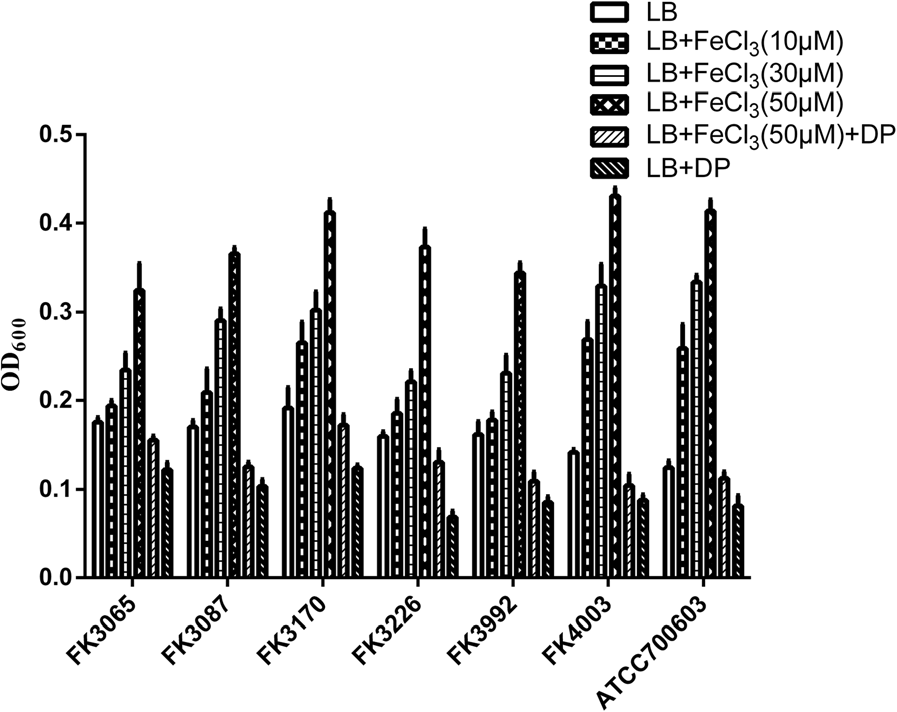
Biofilm-forming capabilities of *K. pneumoniae*. All strains exhibited the strongest biofilm formation ability in LB broth supplemented with 50 μM iron, which was superior to the biofilm formation ability of the respective strains in LB broth containing 30 μM, 10 μM and 0 μM iron or 200 μM iron chelator +(−) 50 μM iron (*P* < 0.05). DP: 2,2′-Dipyridyl, added as an iron chelator. LB + 200 μM iron chelating agent group was used as control

In addition, we further found that iron promotes biofilm formation of *K. pneumoniae* in a concentration-dependent manner. All strains exhibited the strongest biofilm formation ability in LB broth supplemented with 50 μM iron, which was superior to the biofilm formation ability of the respective strains in LB broth containing 30 μM, 10 μM and 0 μM iron (*P* < 0.05). The strains showed a weaker biofilm formation ability in an iron-restricted environment (*P* < 0.05) (Fig. 2).

### The virulence factors of *K. pneumoniae* isolates

The three liver abscess-causing *K. pneumoniae* isolates (FK3226, FK3992 and FK4003) carried at least nine virulence genes, including four kinds of siderophore genes (*entB*, *iucB*, *iroB*, *irp1*). The serotypes of these three isolates were K1, K2 and K1, respectively. The three non-liver abscess-causing *K. pneumoniae* isolates (FK3065, FK3087 and FK3170) carried four virulence genes, including the siderophore gene *entB*, and their serotypes did not belong to one of the common capsule types tested. The virulence genes of each strain were detailed in Table 1.

**Table 1:** The virulence genes of *K. pneumoniae*

Isolates	Virulence genes
FK3065	*entB*, *uge*, *mrkD*, *fimH*
FK3087	*entB*, *uge*, *mrkD*, *fimH*
FK3170	*entB*, *uge*, *mrkD*, *fimH*
FK3226	*iucB*, *iroB*, *entB*, *irp1*, *rmpA*, *wcaG*, *ybtA*, *magA*
	*ureA*, *uge*, *wabG*, *mrkD*
FK3992	*iucB*, *iroB*, *entB*, *irp1*, *rmpA*, *ybtA*, *ureA*, *uge*, *wabG*, *mrkD*
FK4003	*iucB*, *iroB*, *entB*, *irp1*, *rmpA*, *ureA*, *uge*, *wabG*, *mrkD*
ATCC700603	*iucB*, *iroB*, *entB*, *irp1*, *rmpA*, *ybtA*, *ureA*, *wabG*, *mrkD*

### Infection model of *G. mellonella* larvae

**Fig. 3:**
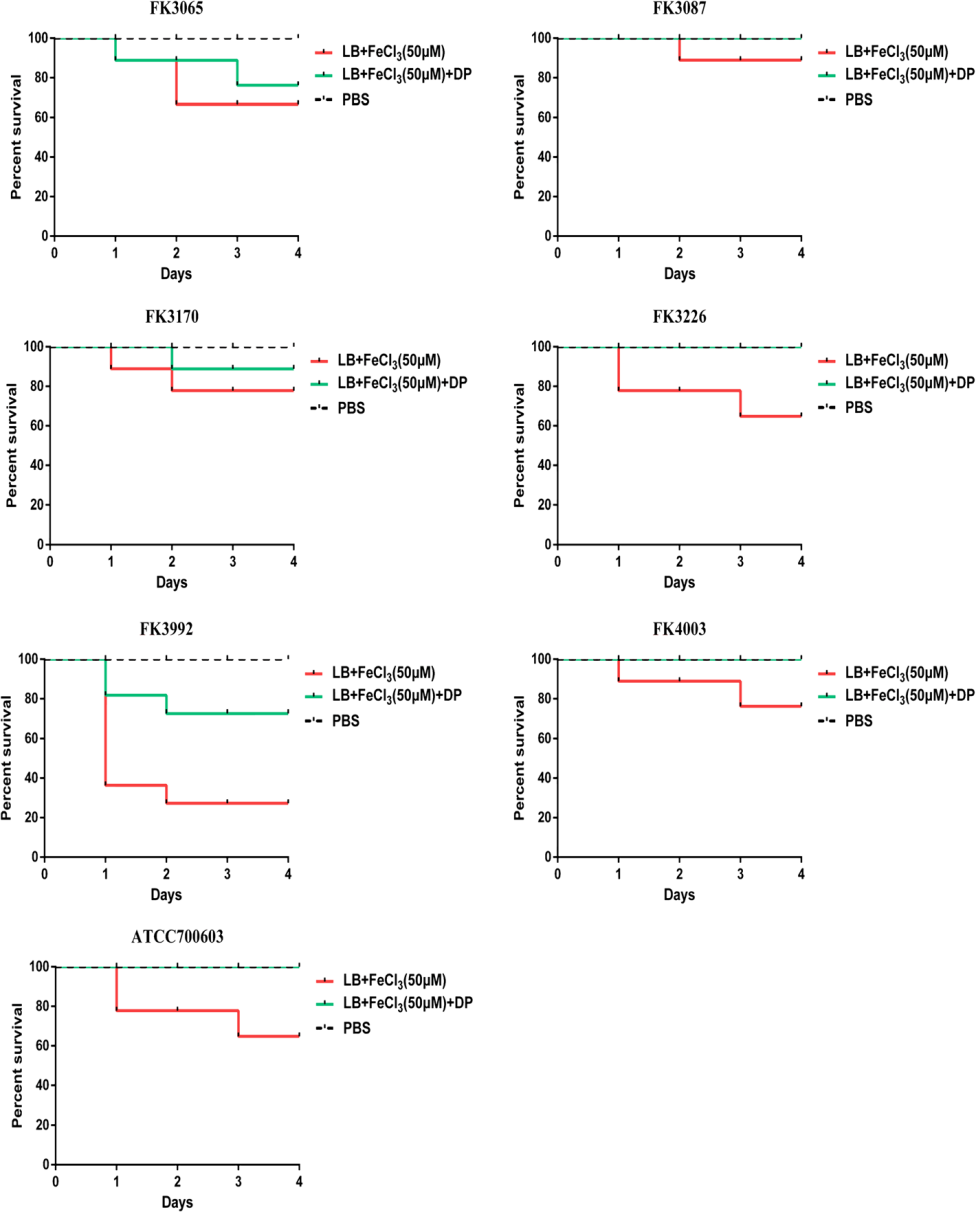
Models of *Galleria mellonella* infected with *K. pneumoniae*. The virulence of FK3226, FK3992 and ATCC 700603 with additional iron was significantly higher than that of the strains with iron chelator (*P* < 0.05). DP: 2,2′-Dipyridyl, added as an iron chelator. The PBS was control group

The mortality rates of *G. mellonella* after infection with the liver abscess-causing *K. pneumoniae* isolates (FK3226, FK3992) and *K. pneumoniae* ATCC700603 cultured with additional iron were significantly higher than those cultured with iron chelator (*P* < 0.05). While the mortality rates of *G. mellonella* after infection with the non-liver abscess-causing *K. pneumoniae* isolates (FK3065, FK3087 and FK3170) and liver abscess-causing *K. pneumoniae* (FK4003) were not significantly different under these two conditions (Fig. 3).

### Quantification of siderophore gene expression with qRT-PCR

**Fig. 4:**
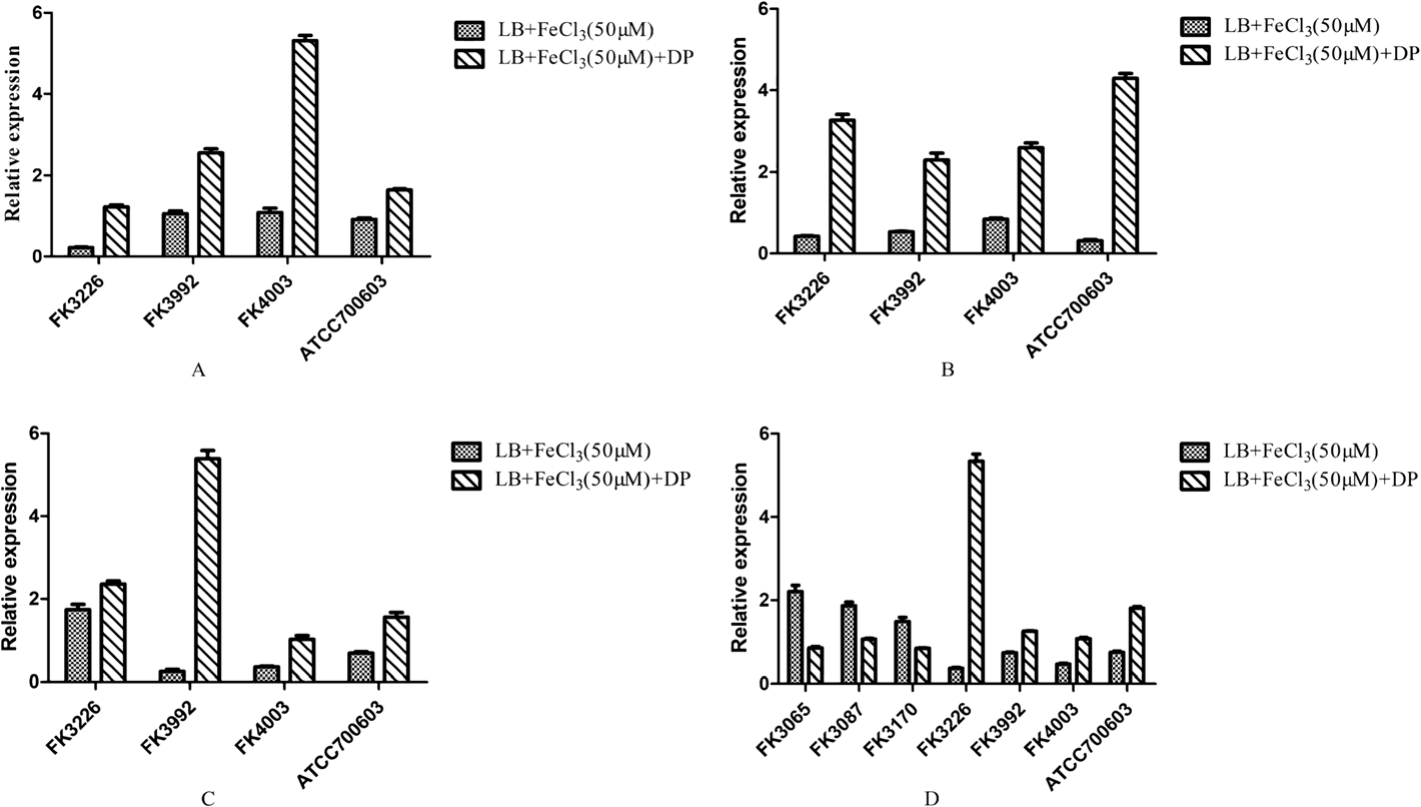
Expression ratio of *K. pneumoniae* virulence genes. **a***iucB* gene. **b***iroB* gene. **c***irp1* gene. **d***entB* gene. DP: 2,2′-Dipyridyl, added as an iron chelator. LB control was used to calculate relative expression

The relative expression levels of the four siderophore genes (*iucB*, *iroB*, *irp1*, *entB*) in the liver abscess-causing *K. pneumoniae* strains cultured with additional iron were lower than those cultured under iron-restricted conditions (*P* < 0.05). On the contrary, the relative expression levels of the siderophore gene *entB* in the non-liver abscess-causing *K. pneumoniae* isolates cultured with extra iron were higher than those cultured under iron-restricted conditions (*P* < 0.05) (Fig. 4).

## Discussion

The Gram-negative bacillus *K. pneumoniae* is a leading cause of healthcare-associated infections, including urinary tract infections, surgical sites infections, soft tissues infections, bacteremia and pneumonia [11]. In the 1980s, case reports from Taiwan described community acquired liver abscesses caused by hvKp in patients with serious concomitant endorgan manifestations, such as meningitis and endophthalmitis [12]. Since the first report in Taiwan, hvKPs have been observed with increasing frequency in many countries in Asia, Europe and America [12]. It is well known that hvKp can invade many organs in patient, including liver. Considering the different iron content between the liver and other tissues, we speculated that this higher iron level may be an important factor to affect the virulence of *K. pneumoniae* in the liver and provide an advantage for liver abscess formation. In this study, three liver abscess-causing *K. pneumoniae* isolates and three non-liver abscess-causing *K. pneumoniae* isolates were selected to perform growth, biofilm and virulence investigations under different iron conditions.

In this research, we demonstrated that moderate amounts of iron promoted the growth of *K. pneumoniae*. A slower growth of *K. pneumoniae* isolates was discovered in iron-restricted environments. Furthermore, it was illustrated that iron promoted biofilm formation of *K. pneumoniae*, and the ability of bacterial biofilm formation was significantly attenuated by addition of an iron chelator. Previously, it has been demonstrated that the biofilm formation ability of *Pseudomonas aeruginosa* was significantly reduced in an iron-restricted environment, and biofilm formation increased significantly after iron complementation, indicating that iron plays an important role in the formation of *P. aeruginosa* biofilms [13]. The presence of an appropriate amount of iron is also conducive to the biofilm formation of *Escherichia coli* and *Staphylococcus aureus* [14, 15]. These findings, together with our investigation, indicate that iron plays a crucial role in the process of bacterial biofilm formation and growth.

In this study, the virulence genes and capsular serotypes of seven experimental strains were detected by PCR. These three liver abscess-causing *K. pneumoniae* isolates carried more virulence genes, including all four siderophore genes *iucB*, *iroB*, *irp1*, *entB*, while the strains isolated from the other sites only carried enterobactin (*entB*) among their four virulence factors. Bacteria secrete siderophores to bind and import iron, which is required for growth. In this investigation, the effect of iron concentration on the relative expression of siderophore genes was detected by qRT-PCR. As expected, the tested *K. pneumoniae* strains causing liver abscesses showed lower relative expression levels of the four siderophore genes (*iucB*, *iroB*, *irp1*, *entB*) in media with high iron levels, compared to iron-restricted media. In the presence of the iron chelator, high-affinity iron binding siderophores are needed to obtain sufficient iron for growth, while at high iron concentrations less metabolically costly uptake mechanisms can be employed. In contrast, the single siderophore gene *entB* of the non-liver abscess-causing *K. pneumoniae* isolates was expressed relatively higher after addition of iron than during iron deficiency caused by iron chelating agent. This is a surprising finding, which might indicate that these strains rely on other iron uptake mechanisms apart from siderophores, which warrants further study.

Previous studies have shown that different concentrations of iron could affect the surface characteristics of the pili and porins and further influence the virulence of *Acinetobacter baumannii* [16]. Therefore, We attempted to demonstrate that this higher iron level may be an important factor to affect the virulence of *K. pneumoniae* in the liver. To investigate the effect of iron on the virulence of *K. pneumoniae*, we determined the virulence of *K. pneumoniae* through the wax larvae infection model. Wax larvae are an invertebrate host model frequently used to detect the virulence of *K. pneumoniae* [17]. According to the results of the *G. mellonella* infection model, three strains caused higher mortality rate with extra iron than iron-restricted growth environment, indicating the potential virulence in vivo. The results of qRT-PCR indicate that a lower expression of siderophore genes correlates with increased virulence. Due to the various needs of different strains for iron, the expression of siderophore genes was not exactly the same. Other mechanisms by which iron affects the virulence of *K. pneumoniae* need to be further explored.

## Conclusions

In summary, iron can promote the growth, biofilm formation and enhance virulence of *K. pneumoniae* causing liver abscess. Moreover, the diverse expression of siderophores genes may be one of the factors that regulate the variation in virulence. To further understand these phenomena, a deeper evaluation of the phenomena is warranted.

## Methods

### Bacterial strains and antimicrobial susceptibility profiling

Six *K. pneumoniae* clinical isolates (FK3065, FK3087, FK3170, FK3226, FK3992, FK4003) were collected from patients in the First Affiliated Hospital of Wenzhou Medical University, Wenzhou, Zhejiang, China during 2016 and 2017. Herein, FK3226, FK3992, FK4003 were isolated from patients with liver abscess, and FK3065, FK3087, FK3170 were isolated from other abdominal invasive infection sites of non-liver abscess patients (ascites, biliary drainage fluid). Identification was conducted on all isolates using VITEK MS system (bioMérieux, Marcy L’Etoile, France). Antimicrobial susceptibility testing was performed by VITEK2 system (bioMérieux, Marcy L’Etoile, France) with AST-GN13 card. *K. pneumoniae* ATCC 700603 served as the control strain.

### Growth curves and biofilm formation

The effect of iron on the growth of *K. pneumoniae* was measured following previous methods with some modifications [18, 19]. In brief, overnight cultures of all *K. pneumoniae* clinical isolates (FK3065, FK3087, FK3170, FK3226, FK3992, FK4003) and *K. pneumoniae* ATCC 700603 were diluted 1:100 in Luria-Bertani (LB) broth supplemented with different iron concentrations (50 μM, 30 μM, 10 μM, 0 μM) and with 200 μM iron chelating agent (2,2′-Dipyridyl) + (−) 50 μM iron, respectively. Wherein, strains under LB + 200 μM iron chelating agent condition were used as control and LB + 200 μM iron chelating agent + 50 μM iron was set to produce an iron-restricted condition. The cultures were incubated at 37 °C with constant shaking at 180 rpm. Samples were collected at 0 h, 2 h, 4 h, 6 h, 8 h, 16 h, 24 h and the absorbance at 600 nm was determined. Each sample was measured in triplicates and averages of absorbance values were used for analysis. The growth of *K. pneumoniae* was evaluated by plotting the values of OD_600_ against time.

The biofilm assay was performed as published with some modifications [20]. Briefly, six *K. pneumoniae* clinical isolates and *K. pneumoniae* ATCC 700603 were grown overnight in LB broth. The overnight cultures were then diluted 1:100 in fresh LB broth supplemented with different iron concentrations (50 μM, 30 μM, 10 μM, 0 μM) and with 200 μM iron chelating agent +(−) 50 μM iron. A total of 100 μL of each dilution were added to a 96-well polystyrene microtiter plate and incubated at 37 °C for 24 h. Wells containing media alone were used as blank. Planktonic cells were removed and the wells were washed twice with sterile water, then the wells were stained with 150 μL 0.1% crystal violet for 10 min and rinsed twice with sterile water. Stained biofilms were solubilized with 95% ethanol and quantified by measuring the OD_600_ using a microplate reader. Each sample was measured in triplicates and averages of absorbance values were used for analysis.

### Detection of virulence genes of isolates

Virulence genes (*magA*, *iucB*, *iroB*, *entB*, *irp1, iroN*, *kfuBC*, *rmpA*, *wcaG, alls*, *ybtA*, *ureA*, *uge*, *wabG*, *fimH* and *mrkD*) and capsular serotypes (K1, K2, K5, K20, K54 and K57) of the six *K. pneumoniae* clinical isolates and *K. pneumoniae* ATCC 700603 were amplified by Polymerase Chain Reaction (PCR). Primers for the aforementioned genes are listed in Supplementary Table S1 [21, 22]. Positive PCR products were sequenced by Beijing Genomics Institute Technology Co. Ltd. (Shanghai, China). Nucleotide sequences were compared using BLAST (http://blast.ncbi.nlm.nih.gov/Blast.cgi).

### Infection model of *Galleria mellonella* larvae

*G. mellonella* killing assays were carried out on the six clinical isolates and *K. pneumoniae* ATCC 700603 as described previously, with minor modifications [17]. Eight larvae weighing between 200 mg–250 mg were randomly selected for each strain. A 10 μL of bacterial suspension (10^8^ CFU/mL) in phosphate-buffered saline (PBS) was injected into the last left proleg using a 25 μL Hamilton precision syringe. The bacterial suspension was prepared by culturing the strains in LB broth containing 50 μM iron, and 50 μM iron with 200 μM iron chelating agent for 24 h. Larvae injected with 10 μl PBS were used as control. The insects were incubated at 37 °C in the dark and observed after 24 h, 48 h and 72 h. Larvae were considered dead when they repeatedly failed to respond to physical stimuli. The primary outcome for the insect model was rapidity and extent of mortality of *G. mellonella* assessed with Kaplan-Meier analysis and log-rank test.

### Quantitative reverse transcription PCR (qRT-PCR)

The effects of iron on the expression levels of *K. pneumoniae* siderophore genes (*iucB*, *iroB*, *entB* and *irp1*) were evaluated using quantitative reverse transcription PCR (qRT-PCR). For RNA extraction, *K. pneumoniae* isolates were grown in fresh LB medium with 50 μM iron, and 50 μM iron with 200 μM iron chelating agent at 37 °C for 24 h. *K. pneumoniae* isolates grown in fresh LB medium were used as control. Total RNA was extracted using a RNeasy Mini Kit (Qiagen, Valencia, CA, USA) according to the manufacturer’s instructions. The extracted RNA samples were stored at − 80 °C. Purified RNA was reverse transcribed into cDNA for qRT-PCR analysis using a cDNA synthesis kit (TaKaRa, Tokyo, Japan) according to the manufacturer’s instructions. Gene expression levels were measured with qRT-PCR using a 7500 RT-PGE system (TOYOBO, Osaka, Japan) and SYBR Green qRT-PCR Kit (TOYOBO) with the specific primers listed in Supplementary Table S2 [23]. The *rpoB* gene was used as an internal control to normalize the data. Each sample was measured in triplicates and averages of Ct values were used for analysis. Gene expression levels were calculated using 2^− △△ Ct^ method.

### Statistical analysis

All experiments were conducted independently with at least two replicates on different days, and results were expressed as mean ± standard deviation or average. The total area under the curve was calculated for analysis on growth. Unpaired or two-tailed paired t-tests were used to evaluate the significance of differences between two groups. One-way analysis of variance (ANOVA) was performed to analyze the significance among more groups. Statistical significance was determined at *P* < 0.05. Statistical analyses were performed using SPSS version 17.0 statistical software.

## Supplementary information

**Additional file 1: Table S1.** Primers used for PCR amplification of virulence genes

**Additional file 2: Table S2.** Primers used for qRT-PCR of siderophore genes

## Data Availability

The datasets used and analysed during the current study available from the corresponding author on reasonable request.

## Abbreviations

PCR: Polymerase Chain Reaction
qRT-PCR: Quantitative Reverse Transcription Polymerase Chain Reaction
