# Supplementary material for: Effects of iron on the growth, biofilm formation and virulence of Klebsiella pneumoniae causing liver abscess
Source: BMC Microbiol. 2020 Feb 18;20:36. doi: 10.1186/s12866-020-01727-5 (PMC7027070; doi:10.1186/s12866-020-01727-5)
Supplement: Supplementary file 1 — Additional file 1: Table S1. Primers used for PCR amplification of virulence genes [file 12866_2020_1727_MOESM1_ESM.docx]

**Table S1.** **Primers used for PCR amplification of virulence genes**

| **Genes** | **Sequence** | **Tm（℃）** |
| --- | --- | --- |
| *magA* | F: 5‘- GGTGCTCTTTACATCATTGC -3’  R: 5’- GCAATGGCCATTTGCGTTAG -3’ | 58 |
| *iroN* | F: 5’- GGCTACTGATACTTGACTATTC -3’ | 58 |
|  | R: 5’- CAGGATACAATAGCCCATAG -3’ |  |
| *kfuBC* | F: 5’- GAAGTGACGCTGTTTCTGGC -3’ | 58 |
|  | R: 5’- TTTCGTGTGGCCAGTGACTC -3’ |  |
| *rmpA* | F: 5’- ACTGGGCTACCTCTGCTTCA -3’ | 58 |
|  | R: 5’- CTTGCATGAGCCATCTTTCA -3’ |  |
| *wcaG* | F: 5’- GGTTGGKTCAGCAATCGTA -3’ | 58 |
|  | R: 5’- ACTATTCCGCCAACTTTTGC -3’ |  |
| *alls* | F: 5’- CCGAAACATTACGCACCTTT -3’ | 58 |
|  | R: 5’- ATCACGAAGAGCCAGGTCAC -3’ |  |
| *ybtA* | F: 5’- ATGACGGAGTCACCGCAAAC -3’ | 55 |
|  | R: 5’- TTACATCACGCGTTTAAAGG -3’ |  |
| *ureA* | F: 5’- GACAAGCTGTTGCTGTTTACC-3’ | 58 |
|  | R: 5’- CGGGTTGTGAACGGTGAC -3’ |  |
| *uge* | F: 5’- GATCATCCGGTCTCCCTGTA -3’ | 53 |
|  | R: 5’- TCTTCACGCCTTCCTTCACT -3’ |  |
| *wabG* | F: 5’- CGGACTGGCAGATCCATATC -3’ | 58 |
|  | R: 5’- ACCATCGGCCATTTGATAGA -3’ |  |
| *fimH* | F: 5’- TGCTGCTGGGCTGGTCGATG -3’ | 62 |
|  | R: 5’- GGGAGGGTGACGGTGACATC -3’ |  |
| *mrkD* | F: 5’- CCACCAACTATTCCCTCGAA -3’ | 43 |
|  | R: 5’- ATGGAACCCACATCGACATT -3’ |  |
| *iucB* | F: 5’- ATGTCTAAGGCAAACATCGT -3’ | 58 |
|  | R: 5’- TTACAGACCGACCTCCGTGA -3’ |  |
| *entB* | F: 5’- ATTTCCTCAACTTCTGGGGC -3’ | 56 |
|  | R: 5’- AGCATCGGTGGCGGTGGTCA -3’ |  |
| *iroB* | F: 5’- GCATAGGCGGATACGAACAT -3’ | 58 |
|  | R: 5’- CACAGGGCAATTGCTTACCT -3’ |  |
| *irp1* | F: 5’- ATGGATAACTTGCGCTTCTC -3’ | 58 |
|  | R: 5’- CGGTATAGCCGACCTTTCTG -3’ |  |
| K1 | F: 5’- GGTGCTCTTTACATCATTGC -3’ | 54 |
|  | R: 5’- GCAATGGCCATTTGCGTTAG -3’ |  |
| K2 | F: 5’- GACCCGATATTCATACTTGACAGAG-3’ | 58 |
|  | R: 5’- CCTGAAGTAAAATCGTAAATAGATGGC -3’ |  |
| K5 | F: 5’- TGGTAGTGATGCTCGCGA -3’ | 58 |
|  | R: 5’- CCTGAACCCACCCCAATC -3’ |  |
| K20 | F: 5’- CGGTGCTACAGTGCATCATT -3’ | 58 |
|  | R: 5’- GTTATACGATGCTCAGTCGC -3’ |  |
| K54 | F: 5’- CATTAGCTCAGTGGTTGGCT -3’ | 58 |
|  | R: 5’- GCTTGACAAACACCATAGCAG -3’ |  |
| K57 | F: 5’- CTCAGGGCTAGAAGTGTCAT -3’ | 58 |
|  | R: 5’- CACTAACCCAGAAAGTCGAG -3’ |  |
