# Supplementary material for: Effects of iron on the growth, biofilm formation and virulence of Klebsiella pneumoniae causing liver abscess
Source: BMC Microbiol. 2020 Feb 18;20:36. doi: 10.1186/s12866-020-01727-5 (PMC7027070; doi:10.1186/s12866-020-01727-5)
Supplement: Supplementary file 2 — Additional file 2: Table S2. Primers used for qRT-PCR of siderophore genes [file 12866_2020_1727_MOESM2_ESM.docx]

**Table S2. Primers used for qRT-PCR of** **siderophore genes**

| **Gene** | **Sequence** | **Reference** |
| --- | --- | --- |
| *iucB* | F: 5’-ATGTCTAAGGCAAACATCGT -3’  R: 5’- TTACAGACCGACCTCCGTGA -3’ | This study |
| *iroB* | F: 5’- ACGACGGCGAACCCATTATT -3’ | This study |
|  | R: 5’- GACTTCACTGGCGGAATCCA -3’ |  |
| *entB* | F: 5’-ATATCCCGGCGAACAAGGTC-3’ | This study |
|  | R: 5’-CGGCGATATTAGCCACCACT -3’ |  |
| *irp1* | F: 5’- CTCAGTGGCAACAACAGTGC -3’ | This study |
|  | R: 5’- GATGGCGCGGTGAATGTTAC -3’ |  |
| *rpoB* | F: 5’- AAGGCGAATCCAGCTTGTTCAGC -3’ | [23] |
|  | R: 5’-TGACGTTGCATGTTCGCACCCATCA -3’ |  |
